# Supplementary material for: Monitoring circulating cell-free HPV DNA in metastatic or recurrent cervical cancer: clinical significance and treatment implications
Source: eLife. 2025 Sep 17;13:RP101887. doi: 10.7554/eLife.101887 (PMC12443474; doi:10.7554/eLife.101887)
Supplement: Supplementary file 1. [file elife-101887-supp1.docx]

**Supplementary Table 1. Primer and probe sequences for ddPCR, fragment sizes, and annealing temperature.**

| **HPV subtype** | **Forward primer** | **Reverse primer** | **Probe** | **Amplicon size** | **Annealing temperature** |
| --- | --- | --- | --- | --- | --- |
| HPV16 | TCCAGCTGGACAAGCAGAAC | CACAACCGAAGCGTAGAGTC | ACAGAGCCCATTACAAT | 88 pb | 60°C |
| HPV18 | AACATTTACCAGCCCGACGA | TCGTCTGCTGAGCTTTCTAC | AACCACAACGTCACACAA | 106 pb | 60°C |
| HPV31 | CGTTACCTTTTGTTGTCAGTGT | GAACAGTTGGGGCACACGA | ACAGAGCACACAAGTAG | 123 pb | 56°C |
| HPV33 | CAGATGAGGATGAAGGCTTGGA | ACTGTTGACACATAAACGAACTG | CTTGTCCATCTGGCC | 119 pb | 56°C |
| HPV58 | CAGACGAGGATGAAATAGGCTTG | ATGTAGTAATTAGCTGTGGCCGG | CTTGTCCATCTGGCC | 70 pb | 56°C |
| HPV66 | CCGTTAACACCGGAGGAAAA | ATGACCCGGTCCATGCATAT | TGAACATAAAAGACGATTTC | 82 pb | 56°C |
