# Supplementary material for: Monitoring circulating cell-free HPV DNA in metastatic or recurrent cervical cancer: clinical significance and treatment implications
Source: eLife. 2025 Sep 17;13:RP101887. doi: 10.7554/eLife.101887 (PMC12443474; doi:10.7554/eLife.101887)
Supplement: Supplementary file 2. [file elife-101887-supp2.docx]

**Supplementary Table 2. HPV cfDNA Levels and Clinical Treatment and Outcomes in the Whole Group of Patients**

| **No.** | **Age** | **IVB/R** | **Sample** | **HPV**  **Type** | **Pathology** | **PD-L1**  **CPS** | **Transfer model** | **Baseline HPV cfDNA** | **Treatment** | **HPV cfDNA** | **Treatment** | **HPV cfDNA** | **Treatment** | **HPV cfDNA** | **Treatment** | **HPV cfDNA** | **D** | **OS(m)** |
| --- | --- | --- | --- | --- | --- | --- | --- | --- | --- | --- | --- | --- | --- | --- | --- | --- | --- | --- |
| 1 | 57 | R | Sequential | 16 | SC | 5 | LR | 4.7 × 10^2^ | (TP+Bev)*6+ICIt*3-SD | 5.2 × 10^4^ | ICIt*16+ICIc*13-SD | 6.3 × 10^4^ | ICIc*5-PD | - | (nab-TP+BIc)*6+BIc*4-PR | - | N | 46.1 |
| 2 | 50 | IVB | Single | 16 | SC | - | LN | 4.7 × 10^2^ | TP*3+RTpm+TP*2-CR | - | Follow up-CR | - | - | - | - | - | N | 42.3 |
| 3 | 48 | IVB | Sequential | 66 | SC | - | LN | 8.5 × 10^2^ | TP*2+RTp+TP*1-PR | 0 | TP*1 | 0 | RTm-CR | 0 | Follow up-PD | - | Y | 39.8 |
| 4 | 51 | IVB | Single | 16 | SC | - | LN | 2.8 × 10^3^ | TP*3+RTpm+TP*2-PD | - | (ICIk+Apatinib)*2-PD | - | Follow up-PD | - | - | - | Y | 16.3 |
| 5 | 46 | IVB | Single | 18 | LCNEC | - | H | 4.0 × 10^3^ | EP*6-PR | - | (ICIk+Anotilib)*3-PD | - | nab-TP*2-PD | - | - | - | Y | 11.1 |
| 6 | 53 | IVB | Sequential | 58 | SC | - | H | 6.6 × 10^3^ | RTpm+BT*1-PR | 1.5 × 10^3^ | BT*4-CR | 0 | TC*2-CR | 0 | TC*1-CR | 0 | N | 19.3 |
| 7 | 57 | IVB | Single | 16 | SC | - | LN | 7.5 × 10^3^ | RTp-PR | - | RTm-CR | - | Follow up-PD | - | - | - | Y | 33.4 |
| 8 | 49 | IVB | Sequential | 16 | SC | - | LN+H | 1.3 × 10^4^ | TP*2-PR | 1.9 × 10^4^ | TP*2+RTp+TP*1-PR | 7.4 × 10^4^ | TP*1+RTm-PD | - | - | - | Y | 12.5 |
| 9 | 67 | R | Sequential | 16 | SC | 60 | LN | 1.7 × 10^4^ | SBRT | 1.2 × 10^4^ | (TP+BIt)*1-PR | 3.6 × 10^3^ | BIt*5-PR | - | BIt*4-PR | - | N | 53.6 |
| 10 | 62 | IVB | Sequential | 18 | SC | 1 | LN+H | 2.0 × 10^4^ | (TP+BIc)*4-PR | 5.3 × 10^2^ | (TP+BIc)*2+BIc*6-PR | 7.1 × 10^3^ | (BIc)*6-PR | - | (BIc)*3-PD | - | N | 22 |
| 11 | 43 | IVB | Sequential | 16 | SC | 20 | LN | 2.8 × 10^4^ | (TP+Bev)*1 | 1.6 × 10^4^ | (TP+Bev)*1+RTp-PR | 4.6 × 10^2^ | RTm-IT-PD | - | (TP+BIt)*4-PR | - | N | 10.2 |
| 12 | 59 | IVB | Sequential | 16 | SC | - | LN+H | 3.0 × 10^4^ | TP*1 | 3.2 × 10^4^ | TP*1-IT-PD+RFA | 0 | (nab-T+ICIs)*1+ICIs*1-PD | 2.0 × 10^3^ | - | - | Y | 16.5 |
| 13 | 38 | IVB | Sequential | 16 | SC | - | LN | 3.9 × 10^4^ | TP*1 | 1.3 × 10^4^ | TP*1+RTpm+TP*3-PR | 5.4 × 10^4^ | TP*1-CR | - | Follow up-PD | - | N | 36.3 |
| 14 | 36 | IVB | Single | 16 | SC | - | LN | 7.3 × 10^4^ | TP*2+RTp+RTm+TP*2-CR | - | Follow up-CR | - | - | - | - | - | N | 38.9 |
| 15 | 49 | R | Sequential | 16 | AC | - | LN+H | 4.2 × 10^4^ | (TP+BIs)*2-PR | 9.3 × 10^5^ | (TP+BIs)*1-PR | 3.8 × 10^4^ | (TP+BIs)*3+RTm-PR | - | BIs*4-PD | - | Y | 50.2 |
| 16 | 42 | IVB | Sequential | 16 | SC | - | LN | 7.0 × 10^4^ | TP*2+RTpm+TP*1-CR | - | Follow up-CR | 9.8 × 10^3^ | Follow up-CR | 1.7 × 10^4^ | Follow up-CR | - | N | 32.8 |
| 17 | 48 | R | Sequential | 16 | SC | 40 | LN | 7.2 × 10^4^ | RTp-PR | 2.4 × 10^2^ | Follow up 303d-PD | 2.2 × 10^3^ | RTm+(TP+ICI*4)-CR | - | Follow up-CR | - | N | 19.1 |
| 18 | 55 | IVB | Sequential | 16 | SC | - | H | 9.0 × 10^4^ | TP*2+RTpm+TP*4-PR | - | Follow up-PR | 1.0 × 10^4^ | Follow up-PR | 8.4 × 10^3^ | Follow up-PR | - | N | 58.9 |
| 19 | 56 | R | Sequential | 16 | SC | 5 | H | 1.8 × 10^5^ | (nab-TC+Bev)*2-PR | 5.7 × 10^2^ | (nab-TC+Bev)*1-PR | 1.7 × 10^4^ | (nab-TC+Bev)*2-PD | - | (nab-TP+Bev+ICIk)*4-PD | - | Y | 88.5 |
| 20 | 34 | IVB | Single | 16 | SC | - | LN+H | 1.8 × 10^5^ | TP*4-PR | - | Dermatomyositis-IT | - | Follow up-PR | - | - | - | N | 78.5 |
| 21 | 54 | IVB | Single | 16 | SC | - | LN+H | 1.9 × 10^5^ | TP*2-PR | - | EBRT+IT | - | Follow up-PD | - | - | - | Y | 23.7 |
| 22 | 55 | IVB | Sequential | 58 | SC | 25 | LN | 4.9 × 10^5^ | (TP+Bev)*2+RTp-IT-PD | 3.4 × 10^6^ | (TP*Bev)*1-PD | 3.7 × 10^5^ | (nab-T+BIt)*1+RTm-PR | - | (nab-T+ICIt)*2+ICIt*2-PR | - | N | 17 |
| 23 | 37 | R | Sequential | 16 | SC | - | LN+H+DSM | 1.7 × 10^6^ | (TP+Bev)*2-PR | 5.6 × 10^4^ | (TP+Bev)*2-PR | 2.0 × 10^4^ | (TP+Bev)*2+Bev*2-PD | - | (Bev+ICIt)*2-SD | - | N | 68.9 |
| 24 | 66 | IVB | Sequential | 58 | SC | - | LN+H | 3.1 × 10^6^ | TP*1 | 1.6 × 10^4^ | TP*1-PR | 9.0 × 10^3^ | RTpm-CR | - | Follow up-PD | - | Y | 12.4 |
| 25 | 61 | R | Sequential | 31 | SC | - | LN+H+DSM | 3.8 × 10^6^ | RTm+(nab-T+ICIk)*1+G*1-PR | 2.1 × 10^6^ | ICIk*1 | 8.5 × 10^5^ | ICI*1+(nab-T+ICIk)*1-PD | - | Follow up-PD | - | Y | 52.1 |
| 26 | 66 | IVB | Sequential | 16 | SC | 70 | LN+H | 7.4 × 10^6^ | (TP+BIt)*6-PR | 1.8 × 10^4^ | RTpm+IT+BT*2-PR | 3.3 × 10^4^ | (BIt)*6-CR | - | ICIt*9-CR | - | N | 17.9 |
| 27 | 36 | IVB | Sequential | 16 | SC | 5 | LN+H | 9.7 × 10^6^ | (TP+BIt)*8-PR | 1.3 × 10^4^ | RTp+IT-PR | 2.9 × 10^5^ | BT*3-PR | 1.5 × 10^3^ | RTm+ICIt*1-CR | - | N | 19 |
| 28 | 53 | IVB | Sequential | 16 | SC | 10 | LN+H+DSM | 7.7 × 10^2^ | TP*4+RTp-PR | 1.9 × 10^4^ | TP*2-PD | 1.2 × 10^4^ | (TP+BIt)*1-PR | 8.5 × 10^2^ | (TP+BIt)*1+SBRT+BIt*2-PD | - | Y | 24.2 |
|  |  |  |  | 33 |  |  | LN+H+DSM | 1.5 × 10^7^ | TP*4+RTp-PR | 2.8 × 10^5^ | TP*2-PD | 6.7 × 10^4^ | (TP+BIt)*1-PR | 0 | (TP+BIt)*1+SBRT+BIt*2-PD | - |  |  |

**Abbreviations**: AC, adenocarcinoma; Bev, bevacizumab; BIc, bevacizumab+immune checkpoint inhibitor-cadonilimab;BIs, Bevacizumab+immune checkpoint inhibitor-sindilizumab; BIt, Bevacizumab+immune checkpoint inhibitor-tislelizumab; BT, brachytherapy; CPS,combined positive score; CR, complete response; D, death; DSM, diffuse serosal metastasis; EBRT, external beam radiation therapy; HM, hematogenous metastasis; ICIc, immune checkpoint inhibitor-cadonilimab; ICIk, Immune checkpoint inhibitor-pembrolizumab; ICIs, Immune checkpoint inhibitor-sindilizumab; ICIt, Immune checkpoint inhibitor-tislelizumab; IVB, First diagnosis of FIGO (International Federation of Gynecology and Obstetric) stage; IT, Interrupt Treatment; LCNEC, large cell neuroendocrine carcinoma; LNM, lymph node metastasis ; LR, local recurrence; nab-TP, nanoparticle albumin-bound paclitaxel/cisplatin; PD, progressive disease; PR, partial response; R, recurrence; RFA, radiofrequency ablation; RTp, radiotherapy for the primary tumor; RTm, Radiotherapy for the metastatic lesion; RTpm, simultaneous radiotherapy of primary and metastatic lesions; SBRT, stereotactic body radiotherapy; SC, squamous cell carcinoma; SD, Stable Disease; TP, paclitaxel/cisplatin; TC, paclitaxel/carboplatin. It should be noted that due to the width limitations of the table, not all treatments for each patient are included in the table.
