# Supplementary material for: Monitoring circulating cell-free HPV DNA in metastatic or recurrent cervical cancer: clinical significance and treatment implications
Source: eLife. 2025 Sep 17;13:RP101887. doi: 10.7554/eLife.101887 (PMC12443474; doi:10.7554/eLife.101887)
Supplement: Supplementary file 3. [file elife-101887-supp3.docx]

**Supplementary Table 3. Univariate analysis of prognostic factors for overall survival (n=28)**

| **Variable** | **No. of** | **Median** | **Univariate analysis** | |
| --- | --- | --- | --- | --- |
|  | **patients** | **OS(m)** | **HR (95% CI)** | **p-value** |
| Age |  |  |  |  |
| ≥ 52 years | 14 | 52.1 | 0.95(0.29-3.12) | 0.927 |
| < 52 years | 14 | 50.2 | 1 |  |
| HPV genotypes |  |  |  |  |
| HPV16+ | 20 | 88.5 | 0.34(0.08-1.43) | 0.052 |
| Non-HPV16+ | 8 | 32 | 1 |  |
| Histological subtype |  |  |  |  |
| Squamous cell carcinoma | 26 | 52.1 | 0.30(0.03-3.47) | 0.098 |
| Non-squamous cell carcinoma | 2 | 30.7 | 1 |  |
| Primary or Recurrence |  |  |  |  |
| Primary IVB stage | 21 | 39.8 | 2.64(0.85-8.22) | 0.056 |
| Recurrence or metastasis | 7 | 88.5 |  |  |
| Pattern of metastasis |  |  |  |  |
| LR or LNM or HM | 16 | 88.5 | 0.46(0.14-1.46) | 0.233 |
| LN + H ± DSM | 12 | 37.2 |  |  |
| Immunotherapy |  |  |  |  |
| Yes | 16 | 52.1 | 1.10(0.35-3.45) | 0.862 |
| No | 12 | - |  |  |
| Targeted therapy |  |  |  |  |
| Yes | 12 | 88.5 | 0.54(0.17-1.67) | 0.277 |
| No | 16 | 39.8 |  |  |
| Median HPV cfDNA |  |  |  |  |
| < 1.6 × 10^4^ | 15 | 39.2 | 1.34(0.43-4.14) | 0.603 |
| ≥ 1.6 × 10^4^ | 13 | 52.1 |  |  |
| Baseline HPV cfDNA |  |  |  |  |
| <4.1 × 10^4^ | 14 | 39.8 | 1.40(0.47-4.16) | 0.535 |
| ≥4.1 × 10^4^ | 14 | 52.1 |  |  |

**Abbreviations:** CI, confidence interval; DSM, diffuse serosal metastasis; HM, hematogenous metastasis; HR, hazard ratio; LNM, lymph node metastasis; LR, local recurrence; OS, overall survival.
